# Can we manipulate the soil microbiome to promote carbon sequestration in croplands?

**DOI:** 10.1371/journal.pbio.3002207

**Published:** 2023-07-12

**Authors:** Noah Fierer, Corinne M. Walsh

**Affiliations:** 1 Department of Ecology and Evolutionary Biology, University of Colorado, Boulder, Colorado, United States of America; 2 Cooperative Institute for Research in Environmental Sciences, University of Colorado, Boulder, Colorado, United States of America; University of California, Davis, UNITED STATES

## Abstract

Manipulating the microbiome of cropland soils has the potential to accelerate soil carbon sequestration, but strategies to do so need to be carefully vetted. Here, we highlight the general steps required to develop, implement, and validate such microbe-based strategies.

## Abstract 2

The microbiome of cropland soils could be manipulated to accelerate soil carbon sequestration. This Perspective suggests how this could be achieved and outlines the general steps required to develop, implement, and validate such microbial-based strategies.

We need new strategies to accelerate rates of carbon sequestration in soil. This is particularly important in croplands, where soil carbon stocks have been depleted from decades of agricultural activities and where efforts to increase soil carbon storage via direct human intervention are most feasible. Increasing rates of soil carbon sequestration in croplands could help reduce atmospheric carbon concentrations, though the magnitude of this potential mitigation strategy is a subject of debate [1,2]. At the same time, the promotion of soil carbon sequestration can have direct benefits to agricultural sustainability given the broad importance of soil organic carbon concentrations to soil health and productivity [1,3]. Thus, accelerating carbon sequestration in croplands can contribute to climate change mitigation at the global scale while also improving food security at the local scale.

Soil carbon sequestration occurs when carbon accumulates in soil more quickly than it leaves soil over time. While there are many approaches that can be used to try to alter this balance in agricultural systems [1,2], including land management strategies (e.g., cover cropping) or the manipulation of crop traits (e.g., rooting depths), we could also increase soil carbon sequestration by directly manipulating the composition of the soil microbiome (i.e., bacteria, archaea, fungi, viruses, and protists [4]). This type of approach, either used singly or in combination with other approaches, is worth pursuing given that microbial activities largely determine the net flow of carbon in soil systems. Soil microbes control the rates at which organic carbon inputs are processed and stabilized either biochemically or via reactions with mineral surfaces [5]. Microbes can also promote the formation of stable soil aggregates that protect soil carbon pools from mineralization and reduce losses of particulate organic carbon from the soil surface via wind and water erosion [6]. At the same time, microbes convert soil organic carbon to forms of carbon that can leave the system in soluble or gaseous forms (most notably CO_2_ and CH_4_).

Since soil carbon dynamics are so strongly influenced by soil microbes, it is feasible that we could accelerate soil carbon sequestration by manipulating the soil microbiome to favor specific taxa or traits. Of course, doing so effectively is not trivial. The soil microbiome is complex and the specific contributions of most soil microbes to carbon dynamics remain undetermined [4]. Likewise, the processes by which soil carbon is stabilized over time and retained in soil are also complex and highly variable across time and space [5]. Biotic and abiotic interactions can mediate outcomes in unexpected ways. As just one example, microbes that produce metabolites that increase organic carbon retention in some soils may decrease organic carbon retention in other soils via “priming” of preexisting organic carbon stocks (i.e., when additional inputs of labile organic carbon accelerate the microbial decomposition of soil organic matter [7]). Despite these multiple layers of complexity, we can identify ways soil microbiomes could be manipulated to promote soil carbon sequestration (Fig 1), acknowledging that the validity of these strategies is uncertain and will likely be strongly context dependent.

**Fig 1:**
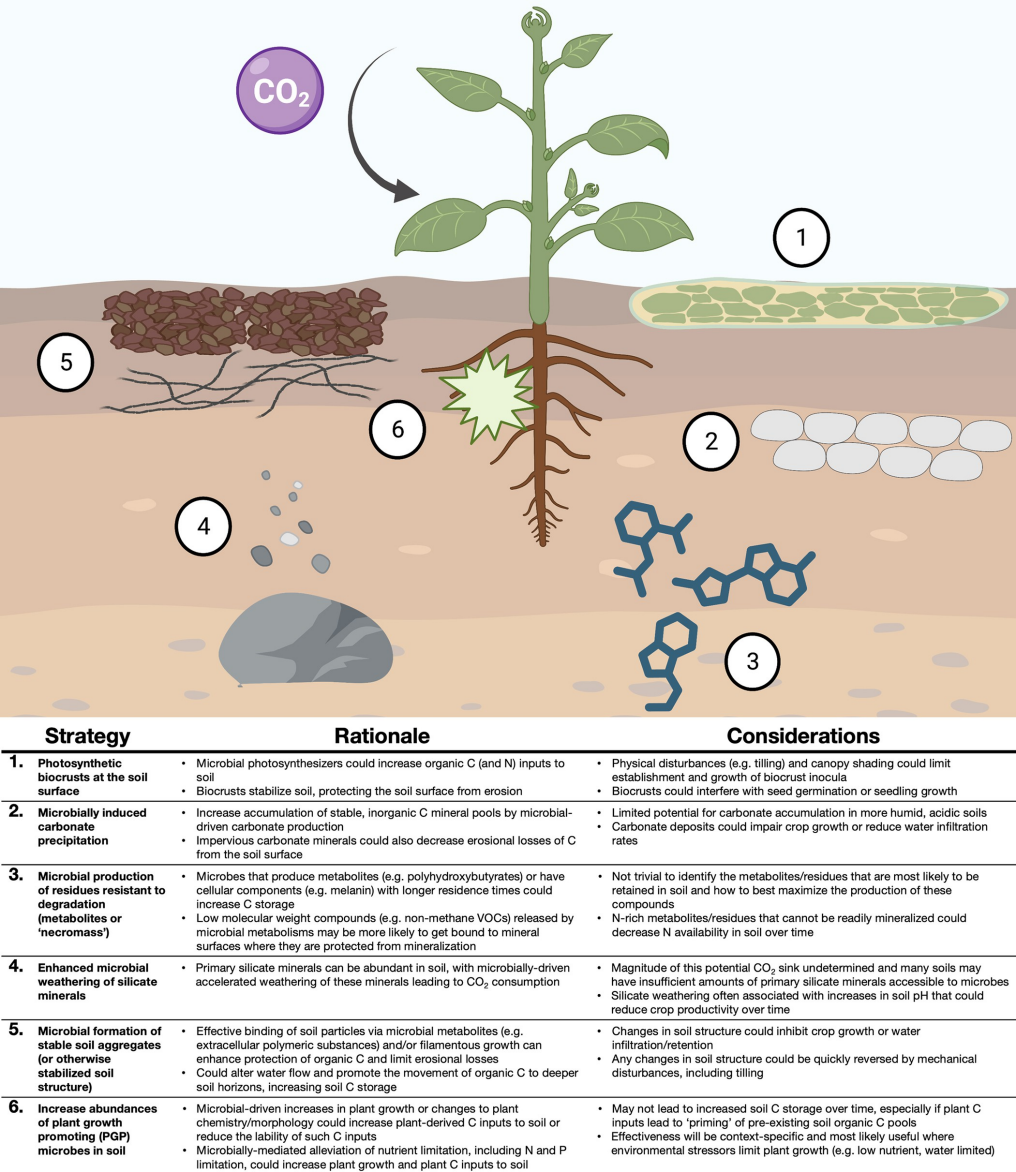
Potential ways the soil microbiome could be manipulated to promote soil carbon sequestration. Six potential strategies for manipulating the soil microbiome to promote soil carbon sequestration and some factors that need to be considered when implementing a given approach. The efficacy of these strategies remains largely undetermined, and the utility of any given strategy will depend strongly on the particular system in question. C, carbon; N, nitrogen; P, phosphorus; VOC, volatile organic compound. Created with BioRender.com.

Regardless of the microbiome-based strategy being explored, there are 3 major steps that have to be followed to develop these strategies, implement them in the field, and validate their effectiveness. The first step is to identify the microbial taxa or microbial traits that may promote soil carbon sequestration (via mechanisms described in Fig 1 or others). This could be done by using *a priori* knowledge of taxa, developing assays to screen for particular traits of interest, or using large databases that couple information from many soil microbiomes with corresponding data on microbial attributes that might increase soil carbon sequestration. For example, as melanin-rich fungal “necromass” is thought to be relatively resistant to decomposition and more likely to be retained in soil over time [8], we could use lab assays or cultivation-independent surveys to identify and better understand the particular taxa with this biochemical trait.

Once potential taxa or traits have been identified, the next step is to figure out how to alter the soil microbiome to increase their abundances. This could be done by adding live microbes directly to soil or seeds. However, this “probiotic” strategy is often limited by the ability to grow sufficient amounts of the microbe(s) of interest and difficulties in getting microbes to successfully establish in the field. Alternatively, we could use a “prebiotic” approach, whereby particular substrates that enrich for the growth of targeted taxa are added as soil amendments, just as prebiotic substrates can be applied as seed coatings to foster plant growth–promoting microbes. Another approach would be to select (or engineer) crop plants to optimize the desired carbon-enhancing plant–microbiome interactions (e.g., mycorrhizal symbioses that enhance soil aggregation) or have tissue or root exudate chemistries that enrich for the targeted microbial taxa or traits. There may also be opportunities to directly manipulate traits in preexisting soil microbial communities via CRISPR gene editing [9], although this technology is still in its infancy. Despite these potential avenues, we emphasize that soil microbiome engineering efforts face considerable challenges—it is not trivial to identify the relevant taxa or traits as most soil microbial taxa remain uncharacterized [4], we do not necessarily know which specific microbial traits are most likely to promote soil carbon sequestration, and we know even less about how to consistently manipulate microbial communities to maintain desired outcomes in the field.

Regardless of how the soil microbiome is altered, the final step is to confirm that the microbiome manipulation actually leads to consistent increases in soil carbon sequestration rates. Accurately measuring or modeling soil carbon sequestration is not easy to do, as soil carbon stocks are often highly heterogeneous across space, even within a given field, and it may take decades for changes in total soil carbon pools to become evident [10]. Moreover, croplands across the globe are facing the consequences of climate change, including changes in temperature and precipitation regimes that need to be accounted for when testing the longer-term efficacy of carbon sequestration strategies.

Any claim that soil carbon sequestration can be increased via manipulation of the soil microbiome should be considered with an abundance of skepticism, given the challenges associated with quantifying longer-term changes in soil carbon sequestration rates under field-relevant conditions. Even well-reasoned hypotheses or expectations may not survive encounters with the complex realities of the soil system. There is not only the risk of wasting time and money implementing strategies that are ultimately unsuccessful but also the risk that microbiome manipulation can have unanticipated and undesirable consequences, including potential reductions in crop yields or introductions of invasive taxa that could proliferate and affect ecosystem health. There is ample motivation to develop new microbial-based soil carbon sequestration strategies and the tools we have at our disposal makes it increasingly feasible to do so, but such strategies need to be carefully vetted before they are widely implemented.
